# Xiao Chai Hu Decoction ameliorates doxorubicin-induced cardiotoxicity through an *Akkermansia muciniphila*–associated gut–heart mechanism

**DOI:** 10.1186/s13020-026-01531-9

**Published:** 2026-11-14

**Authors:** Feixiang Zhai, Linxuan Tao, Yunpu Xia, Qian He, Hui Lin, Dan Liu

**Affiliations:** 1 Jiangxi Provincial Key Laboratory of Drug Target Discovery and Validation, School of Pharmacy, Jiangxi Medical College, Nanchang University, Nanchang, 330031 People’s Republic of China; 2 The MOE Basic Research and Innovation Center for the Targeted Therapeutics of Solid Tumors, Jiangxi Medical College, Nanchang University, Nanchang, China; 3 Queen Mary School, Jiangxi Medical College, Nanchang University, Nanchang, Jiangxi 330031 People’s Republic of China; 4 School of Basic Medical Sciences, Jiangxi Medical College, Nanchang University, Nanchang, 330006 China

**Keywords:** Xiao Chai Hu Decoction, Doxorubicin cardiotoxicity, Ferroptosis, Gut microbiota, *Akkermansia muciniphila*, BMP10

## Abstract

**Background:**

Doxorubicin (DOX) induces cardiotoxicity partly through ferroptosis. Xiao Chai Hu Decoction (XCHD) has anti-inflammatory and organ-protective activities, but its effects on DOX-induced cardiac injury and the gut–heart axis remain unclear.

**Purpose:**

This study investigated whether XCHD attenuates DOX-induced cardiotoxicity through gut microbiota remodeling, with particular emphasis on the contribution of *Akkermansia muciniphila* and the downstream involvement of BMP10-associated ferroptosis.

**Methods:**

Male C57BL/6 mice received DOX (5 mg/kg/week for 4 weeks; cumulative dose, 20 mg/kg) and XCHD (5–20 g/kg/day). Cardiac function, myocardial injury, histopathology, iron accumulation, and ferroptosis-related indices were evaluated. Cardiac transcriptomics and fecal 16S rRNA sequencing were combined with antibiotic-mediated microbiota depletion, fecal microbiota transplantation (FMT), and *A. muciniphila* supplementation to assess microbiota involvement. H9c2 cells treated with XCHD-containing serum and subjected to BMP10 knockdown were used to examine the downstream role of BMP10.

**Results:**

XCHD improved cardiac function and reduced myocardial injury, fibrosis, iron accumulation, and ferroptosis-associated alterations. Transcriptomic analysis identified BMP10 as a candidate downstream factor suppressed by DOX and restored by XCHD. In H9c2 cells, XCHD-containing serum attenuated ferroptosis-associated changes, whereas BMP10 knockdown weakened these effects, supporting a functional contribution of BMP10 in vitro. XCHD also preserved intestinal barrier integrity, reshaped the gut microbiota, and enriched *A. muciniphila*. Microbiota depletion abolished the protective effects of XCHD, whereas FMT transferred the protective phenotype. *A. muciniphila* supplementation reproduced key cardiac and anti-ferroptotic effects, with live bacteria producing greater benefits than heat-inactivated bacteria; these changes were accompanied by restoration of myocardial BMP10 expression.

**Conclusion:**

XCHD alleviates DOX-induced cardiotoxicity through an *A. muciniphila*-associated, microbiota-dependent gut–heart mechanism involving intestinal barrier preservation and attenuation of ferroptosis-associated injury. These findings support a “TCM formula–gut microbiota–host response–regulated cell death” framework for XCHD-mediated cardioprotection.

**Supplementary Information:**

The online version contains supplementary material available at https://doi.org/10.1186/s13020-026-01531-9.

## Introduction

Doxorubicin (DOX) remains a cornerstone chemotherapeutic agent for solid tumors and hematological malignancies, but its clinical use is limited by cumulative cardiotoxicity that may progress to irreversible cardiomyopathy and heart failure[1–3]. DOX-induced cardiotoxicity (DIC) involves oxidative stress, calcium dysregulation, mitochondrial dysfunction, and multiple forms of regulated cell death[4–7]. Among these, ferroptosis, characterized by iron-dependent lipid peroxidation, has emerged as an important contributor to DIC. DOX-induced iron dyshomeostasis, glutathione peroxidase 4 (GPX4) inactivation, and mitochondrial lipid peroxidation promote ferroptotic cardiomyocyte injury, whereas ferroptosis inhibition confers cardioprotection in preclinical models[8]. Thus, targeting ferroptosis represents a promising strategy for preventing DIC.

Traditional Chinese medicine (TCM) has shown potential in mitigating DIC through its multicomponent and multitarget properties. Previous studies have mainly focused on isolated compounds or cardiomyocyte-intrinsic pathways, including nuclear factor erythroid 2–related factor 2/heme oxygenase−1 (Nrf2/HO-1), phosphoinositide 3-kinase/protein kinase B (PI3K/Akt), and nuclear factor kappa B (NF-κB) signaling pathways[9, 10], whereas systemic host–microbiota interactions remain less understood. This gap is particularly relevant because DOX disrupts intestinal barrier integrity and gut microbial homeostasis, thereby promoting systemic inflammation, metabolic disturbance, and cardiac injury through the gut–heart axis[11, 12]. Moreover, the extensive interactions between orally administered herbal constituents and the gut microbiota may contribute substantially to the systemic effects of TCM formulas[13, 14].

Xiao Chai Hu Decoction (XCHD) is a classical formula traditionally used to harmonize Shaoyang and coordinate systemic functional balance, a concept compatible with the modern view of microbiota-mediated interorgan communication. Pharmacologically, XCHD exerts anti-inflammatory, antioxidant, and immunomodulatory effects and has been reported to regulate hepatic immune responses, modulate intestinal microbiota, and alleviate sepsis-induced myocardial injury[15–17]. These findings provide both an organ-protective and microbiota-related basis for investigating XCHD in DIC.

Among the intestinal bacteria implicated in host metabolic and immune homeostasis, *Akkermansia muciniphila* (AKK) is recognized for its ability to preserve intestinal barrier integrity and modulate systemic inflammatory and metabolic responses. Beyond its established role in cardiac development, bone morphogenetic protein 10 (BMP10) has recently been linked to adult myocardial iron homeostasis, with BMP10 deficiency promoting cardiac iron accumulation[18]. These observations raise the possibility that microbiota-associated gut–heart signaling may influence myocardial ferroptosis through changes in BMP10 expression and iron homeostasis.

However, whether XCHD protects against DIC through a microbiota-dependent gut–heart mechanism involving AKK, myocardial BMP10, and ferroptosis-associated injury remains unknown. In particular, it has not been determined whether XCHD-induced enrichment of AKK is associated with restoration of myocardial BMP10 expression, maintenance of iron homeostasis, and attenuation of ferroptosis-associated injury. We therefore hypothesized that XCHD alleviates DIC by reshaping the gut microbiota and enriching AKK, thereby generating gut–heart signals that favor myocardial BMP10 restoration and attenuate ferroptosis-associated injury. To test this hypothesis, we combined cardiac transcriptomics, fecal 16S ribosomal RNA (rRNA) gene sequencing, antibiotic-mediated microbiota depletion, fecal microbiota transplantation (FMT), AKK supplementation, and BMP10 loss-of-function analysis.

## Materials and methods

### Preparation of XCHD and XCHD-containing serum

XCHD consisted of *Bupleurum chinense* DC (24 g), *Scutellaria baicalensis* Georgi (9 g), *Panax ginseng* C. A. Mey. (9 g), *Glycyrrhiza uralensis* Fisch. (9 g), *Zingiber officinale* Roscoe (9 g), *Pinellia ternata* (Thunb.) Makino (9 g), and *Ziziphus jujuba* Mill. (12 g). The decoction was prepared as previously described[17]. Briefly, the herbs were decocted twice with water, filtered, and concentrated to 2.5 g/mL. Male Sprague–Dawley rats received XCHD (7.3 g/kg) by oral gavage once daily for 7 days, and blood was collected 2 h after the final administration according to established serum pharmacology protocols[19–21]. The dose was selected based on body surface area normalization of the human reference dose of 81 g crude herbs per day for a 60-kg adult and was comparable to the 7.02 g/kg dose previously used for XCHD-containing serum preparation in Sprague–Dawley rats[22, 23]. The serum was separated, sterile-filtered, and stored at −80 °C. The chemical profiles of the XCHD decoction and XCHD-containing serum were characterized using our previously established ultra-performance liquid chromatography–tandem mass spectrometry (UPLC–MS/MS) method[17]. Thirty-one compounds detected in both preparations are listed in Supplementary Table S1.

### AKK preparation and administration

AKK (ATCC BAA-835) was cultured anaerobically at 37 °C in brain heart infusion broth supplemented with 0.5% (w/v) porcine gastric mucin. Bacterial cells were harvested at the mid-logarithmic phase (optical density at 600 nm [OD₆₀₀] = 0.8–1.0), washed twice with pre-reduced sterile phosphate-buffered saline (PBS), and resuspended in the same buffer. The bacterial concentration was estimated using an OD₆₀₀ calibration curve, verified by anaerobic plate counting, and adjusted to 1.25 × 10⁸ colony-forming units (CFU)/mL. Heat-inactivated AKK (HI-AKK) was prepared by incubation at 70 °C for 30 min, and complete inactivation was confirmed by the absence of bacterial growth after 72 h of anaerobic culture. Live AKK or HI-AKK was administered by oral gavage at a dose equivalent to 1 × 10⁹ CFU/kg per administration once every 3 days. The gavage volume was adjusted according to body weight. Fresh live bacterial suspensions were administered within 2 h of preparation to minimize oxygen exposure and preserve bacterial viability. Pre-reduced sterile PBS was used as the vehicle control.

### Animal experimental design

**Fig. 1:**
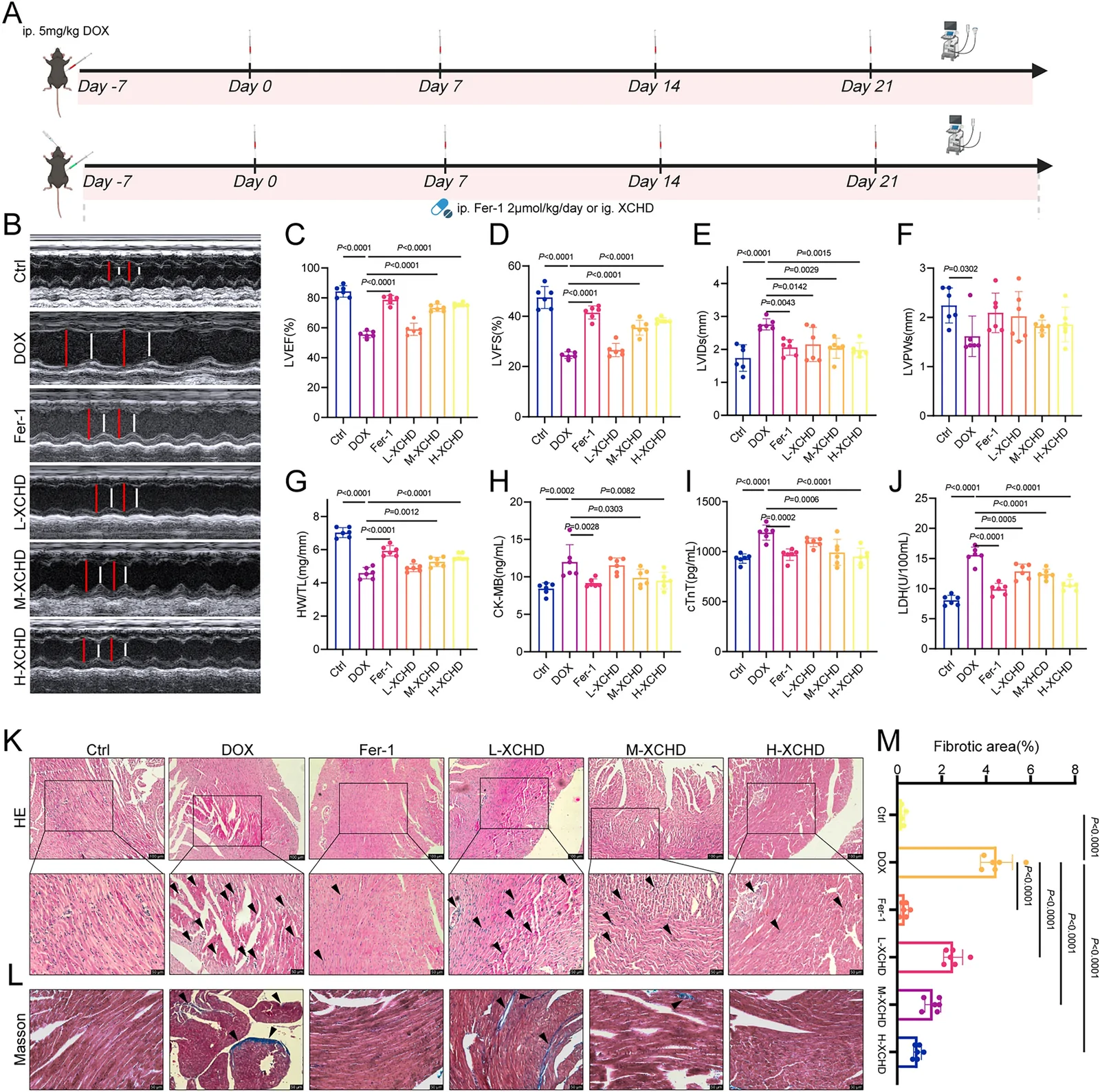
XCHD ameliorates DOX-induced cardiac dysfunction and structural remodeling in mice. **A** Experimental design and treatment schedule. Mice received weekly intraperitoneal DOX (5 mg/kg) for four weeks, with concurrent daily administration of XCHD (low, medium, or high dose) or Fer-1. **B** Representative M-mode echocardiographic images of the left ventricle. **C**–**F** Quantitative analysis of echocardiographic parameters: LVEF (**C**), LVFS (**D**), LVIDs (**E**), and LVPWs (**F**). **G** HW/TL ratio. **H**–**J** Serum levels of myocardial injury markers: CK-MB (**H**), cTnT (**I**), and LDH (**J**). **K** Representative H&E staining of myocardial tissue (scale bar = 50 μm). **L** Representative Masson's trichrome staining showing collagen deposition (scale bar = 50 μm). **M** Quantification of myocardial fibrotic area. Data are expressed as mean ± SD (n = 6). Statistical analysis was performed using one-way ANOVA followed by Dunnett's post hoc test. *P* values are indicated in the figures

Male C57BL/6 mice (6–8 weeks old) were randomly assigned to six groups: Control (saline), DOX (A1832, APEXBIO), DOX + ferrostatin-1 (Fer-1; T6500, TargetMol), DOX + low-dose XCHD (L-XCHD; 5 g/kg/day), DOX + medium-dose XCHD (M-XCHD; 10 g/kg/day), and DOX + high-dose XCHD (H-XCHD; 20 g/kg/day). The XCHD doses were derived from the adult clinical dose by body surface area conversion[22]. Chronic DOX-induced cardiotoxicity was established by intraperitoneal injection of DOX (5 mg/kg) once weekly for 4 consecutive weeks, resulting in a cumulative dose of 20 mg/kg[24, 25]. Fer-1 (2 μmol/kg/day) was administered intraperitoneally throughout the experimental period, whereas XCHD was administered by oral gavage once daily at the indicated doses. The detailed experimental schedule is shown in Fig. 1A.

To establish a pseudo-germ-free (PGF) phenotype, mice received a broad-spectrum antibiotic (ABX) cocktail containing metronidazole (T1079, TargetMol), ampicillin (T0814L, TargetMol), vancomycin (T8641, TargetMol), and neomycin sulfate (T0950, TargetMol) by oral gavage for 1 week. The mice were then assigned to two groups: ABX-DOX and ABX-XCHD. DOX-induced cardiotoxicity was established as described above, and mice in the ABX-XCHD group additionally received XCHD (20 g/kg/day) by oral gavage during the DOX treatment period.

For the FMT experiment, recipient mice were divided into two groups: FMT-Ctrl and FMT-XCHD. Fresh feces collected from saline- or XCHD-treated donor mice were resuspended in pre-cooled sterile saline at a ratio of 1:10 (w/v). After vortex homogenization, the suspension was centrifuged at 3,000 rpm for 10 min at 4 °C. The resulting supernatant was adjusted to a uniform bacterial concentration, and recipient mice received 0.2 mL of the fecal suspension by oral gavage according to the schedule shown in Fig. 6A.

For the AKK supplementation experiment, mice were divided into four groups: ABX-Ctrl, ABX-DOX, ABX-HI-AKK, and ABX-AKK. Following establishment of the pseudo-germ-free phenotype, DOX-induced cardiotoxicity was established as described above. During DOX treatment, mice in the ABX-HI-AKK and ABX-AKK groups received heat-inactivated or live AKK, respectively, at 1 × 10⁹ CFU/kg per administration by oral gavage once every 3 days. Before each administration, bacterial concentrations were confirmed by plate counting, and the viability of live AKK was verified by anaerobic culture. Bacterial suspensions were prepared and administered rapidly under aseptic conditions to minimize oxygen exposure.

Eight mice were initially assigned to each group to account for potential attrition during the experimental period. Six biological replicates per group were used for most in vivo endpoint analyses; when more than six animals were available, six were randomly selected before outcome assessment. The exact number of biological replicates included in each analysis is specified in the corresponding figure legend.

All mice were housed under specific pathogen-free conditions at 22 ± 2 °C and 50 ± 5% relative humidity under a 12-h light/dark cycle, with free access to standard chow and sterile water. All animal procedures were approved by the Animal Ethics Committee of Nanchang University (Approval No. NCULAE−20241218001; approval date: December 19, 2024).

### Echocardiography

Cardiac function was assessed by transthoracic echocardiography as previously described[17]. Briefly, mice were anesthetized with isoflurane, and M-mode images were acquired at the papillary muscle level to determine left ventricular ejection fraction (LVEF), left ventricular fractional shortening (LVFS), left ventricular internal diameter at end systole (LVIDs), and left ventricular posterior wall thickness at end systole (LVPWs), with all measurements performed by investigators blinded to the experimental group assignments.

### Detection of serum myocardial injury factors

Mice were anesthetized, and whole blood was collected from the abdominal aorta. Serum was obtained by centrifugation after clotting at room temperature and used to measure creatine kinase-MB (CK-MB, ED-27926, LunChangShuoBiotech), cardiac troponin T (cTnT, ED-20717, LunChangShuoBiotech), and lactate dehydrogenase (LDH, BB-47214, BestBio) using commercial assay kits according to the manufacturers' instructions. Absorbance was recorded with a microplate reader (PerkinElmer, USA), and analyte concentrations were calculated from standard curves.

### Histological analyses

Histological staining kits included hematoxylin and eosin (H&E; G1120, Solarbio), Alcian blue–periodic acid–Schiff (AB–PAS; G1285, Solarbio), modified Masson’s trichrome staining (G1346, Solarbio), and Prussian blue iron staining (G1428, Solarbio). Paraffin-embedded colon sections were stained with H&E and AB–PAS to evaluate histological morphology, inflammatory infiltration, mucosal integrity, and mucin secretion. Paraffin-embedded heart sections were subjected to H&E, Masson’s trichrome, and Prussian blue staining to evaluate myocardial histopathology, fibrosis, and iron deposition, respectively, according to previously reported protocols[11, 26]. Histological images were acquired and quantitatively analyzed by investigators blinded to the experimental group assignments using a Leica DMi8 microscope.

### Cardiac Fe^2^⁺ detection

The ferrous ion assay kit (BC5415, Solarbio) was used to determine Fe^2^⁺ levels in cardiac tissue following the manufacturer’s instructions. Briefly, myocardial tissues were homogenized with the kit extraction buffer on ice, followed by centrifugation to obtain supernatants. After color reaction at room temperature in the dark, the absorbance at 593 nm was determined using a microplate reader (PerkinElmer, USA). Tissue Fe^2^⁺ content was calculated based on the standard curve and normalized to tissue wet weight.

### Transcriptomic and 16S rRNA gene sequencing

Cardiac transcriptomic profiling and fecal 16S rRNA gene sequencing were performed by Metware Biotechnology Co., Ltd. (Wuhan, China). For transcriptomic analysis, total RNA was extracted from myocardial tissues using TRIzol reagent and assessed using a Qubit fluorometer and Qsep400 biofragment analyzer. Qualified libraries were sequenced on an Illumina platform to generate 150-bp paired-end reads. Clean reads were aligned to the mouse reference genome using HISAT2, gene-level counts were generated using featureCounts, and differential expression analysis was performed using DESeq2, followed by Gene Ontology (GO) and Kyoto Encyclopedia of Genes and Genomes (KEGG) enrichment analyses.

For 16S rRNA gene sequencing, microbial DNA was extracted from four independent fecal samples per group using the cetyltrimethylammonium bromide (CTAB) method. The V4 region was amplified using primers 515F/806R and sequenced on an Illumina NovaSeq platform to generate 250-bp paired-end reads. After quality filtering, read merging, and chimera removal, sequences were clustered into operational taxonomic units (OTUs) at 97% similarity using UPARSE and annotated against the SILVA database. Alpha diversity, beta diversity, and principal coordinates analysis (PCoA) were analyzed using Quantitative Insights Into Microbial Ecology (QIIME).

### Western blot

Total protein was extracted from tissues and H9c2 cells using radioimmunoprecipitation assay (RIPA) lysis buffer (R0020, Solarbio) and quantified using a bicinchoninic acid (BCA) assay. Equal amounts of protein were separated by sodium dodecyl sulfate–polyacrylamide gel electrophoresis (SDS–PAGE) and transferred to polyvinylidene difluoride (PVDF) membranes. After blocking with 5% skim milk for 2 h at room temperature, the membranes were incubated overnight at 4 °C with primary antibodies against PTGS2 (ET1610-23, HUABIO; 1:1,000), BMP10 (163,282, Zenbio; 1:1,000), GPX4 (ET1706-45, HUABIO; 1:4,000), ZO-1 (21,773–1-AP, Proteintech; 1:500), occludin (27,260–1-AP, Proteintech; 1:2,000), claudin-5 (29,767–1-AP, Proteintech; 1:2,000), and GAPDH (60,004–1-Ig, Proteintech; 1:8,000). After washing with Tris-buffered saline containing Tween 20 (TBST), the membranes were incubated with secondary antibodies (1:4,000) for 2 h at room temperature. Protein bands were visualized using enhanced chemiluminescence (ECL). All western blot experiments were performed using three independent biological replicates. Band intensities were quantified using ImageJ software, normalized to GAPDH, and subjected to semiquantitative analysis.

### Cell culture

H9c2 cells were cultured in high-glucose Dulbecco’s modified Eagle’s medium (DMEM) containing 10% fetal bovine serum (FBS) and 1% penicillin–streptomycin (100 U/mL penicillin and 100 μg/mL streptomycin) and passaged at 80–90% confluence. Normal serum (NS) from saline-treated rats served as the control serum. Cells were divided into six groups: NS, DOX, DOX + Fer-1 (1 μM), and DOX plus low-, medium-, or high-dose XCHD-containing serum (DCS; 0.5%, 1%, and 2% [v/v], respectively). The final serum concentration was maintained at 2% (v/v) in all groups by supplementing NS as needed. Following 24 h of pretreatment, all groups except the NS group were challenged with DOX (1 μM) for 12 h.

### Cell viability assay

H9c2 cells were seeded in 96-well plates and pretreated with DCS or Fer-1 for 24 h, followed by DOX exposure for 12 h. Cell viability was determined using the Cell Counting Kit-8 (CCK-8) assay (GK10001, GLPbio). Absorbance at 450 nm was measured using a microplate reader (PerkinElmer, USA), and cell viability was calculated relative to the control group.

### Measurement of oxidative stress markers

Cardiac tissue samples and H9c2 cell lysates were prepared for the detection of oxidative stress biomarkers. Malondialdehyde (MDA) levels and the ratio of reduced glutathione (GSH) to oxidized glutathione (GSSG) were determined using the corresponding commercial assay kits (MDA, BC6415, Solarbio; GSH/GSSG, A061-1-2, Nanjing Jiancheng Bioengineering Institute) according to established protocols[25].

### Lipid ROS and intracellular Fe^2+^ detection

Lipid reactive oxygen species (ROS) were detected using the C11-BODIPY 581/591 probe (HY-D1301, MedChemExpress). Intracellular Fe^2^⁺ was detected using the FerroOrange fluorescent probe (F374, Dojindo). Following drug treatment, cells were incubated with the C11-BODIPY 581/591 or FerroOrange probe for 30 min at 37 °C and imaged using a laser scanning confocal microscope (Leica STELLARIS 5), as described previously[27].

### Statistical analysis

Data are presented as the mean ± standard deviation (SD). Statistical analyses were performed using GraphPad Prism version 10.6.1 (GraphPad Software, Boston, MA, USA). Comparisons among more than two groups were performed using one-way analysis of variance (ANOVA) followed by Dunnett’s post hoc test, whereas comparisons between two groups were performed using an unpaired Student’s t test. Correlations were evaluated using Pearson correlation analysis. All in vitro experiments were performed in at least three independent experiments. Statistical significance was defined as *P* < 0.05, and numerical *P* values are shown in the figure panels.

## Results

### Xiao Chai Hu Decoction ameliorates DOX-induced cardiac dysfunction and structural remodeling

A murine model of DOX-induced cardiotoxicity was established by weekly intraperitoneal administration of DOX (5 mg/kg) for four consecutive weeks, with concurrent oral administration of XCHD at different doses (Fig. 1A). Echocardiographic analysis showed that DOX impaired left ventricular systolic function, as evidenced by significant reductions in LVEF and LVFS, accompanied by an increase in LVIDs and a decrease in LVPWs (Fig. 1B-F). XCHD treatment attenuated these functional abnormalities in a dose-dependent manner. Macroscopic assessment further showed cardiac atrophy in DOX-treated mice, as reflected by a significant reduction in the heart weight-to-tibial length ratio (HW/TL). XCHD treatment attenuated the DOX-induced reduction in the HW/TL ratio in a dose-dependent manner (Fig. 1G).

Consistent with the functional and morphological findings, biochemical analyses showed that DOX administration induced myocardial injury, as indicated by increased serum levels of CK-MB, cTnT, and LDH. These changes were significantly attenuated by XCHD treatment (Fig. 1H–J). Histopathological examination further supported the cardioprotective effects of XCHD. Representative H&E-stained sections showed myocardial fiber disorganization and widened interstitial spaces in DOX-treated hearts (Fig. 1K), whereas Masson’s trichrome staining revealed increased collagen deposition and myocardial fibrosis (Fig. 1L). XCHD administration preserved myocardial architecture and attenuated collagen deposition. Quantitative analysis of the fibrotic area further confirmed that XCHD significantly reduced myocardial fibrosis compared with the DOX group (Fig. 1M). Collectively, these findings indicate that XCHD attenuates DOX-induced cardiac dysfunction, myocardial injury, cardiac atrophy, and structural remodeling.

### Xiao Chai Hu Decoction attenuates DOX-induced ferroptosis-associated alterations in vivo and restores myocardial BMP10 expression

**Fig. 2:**
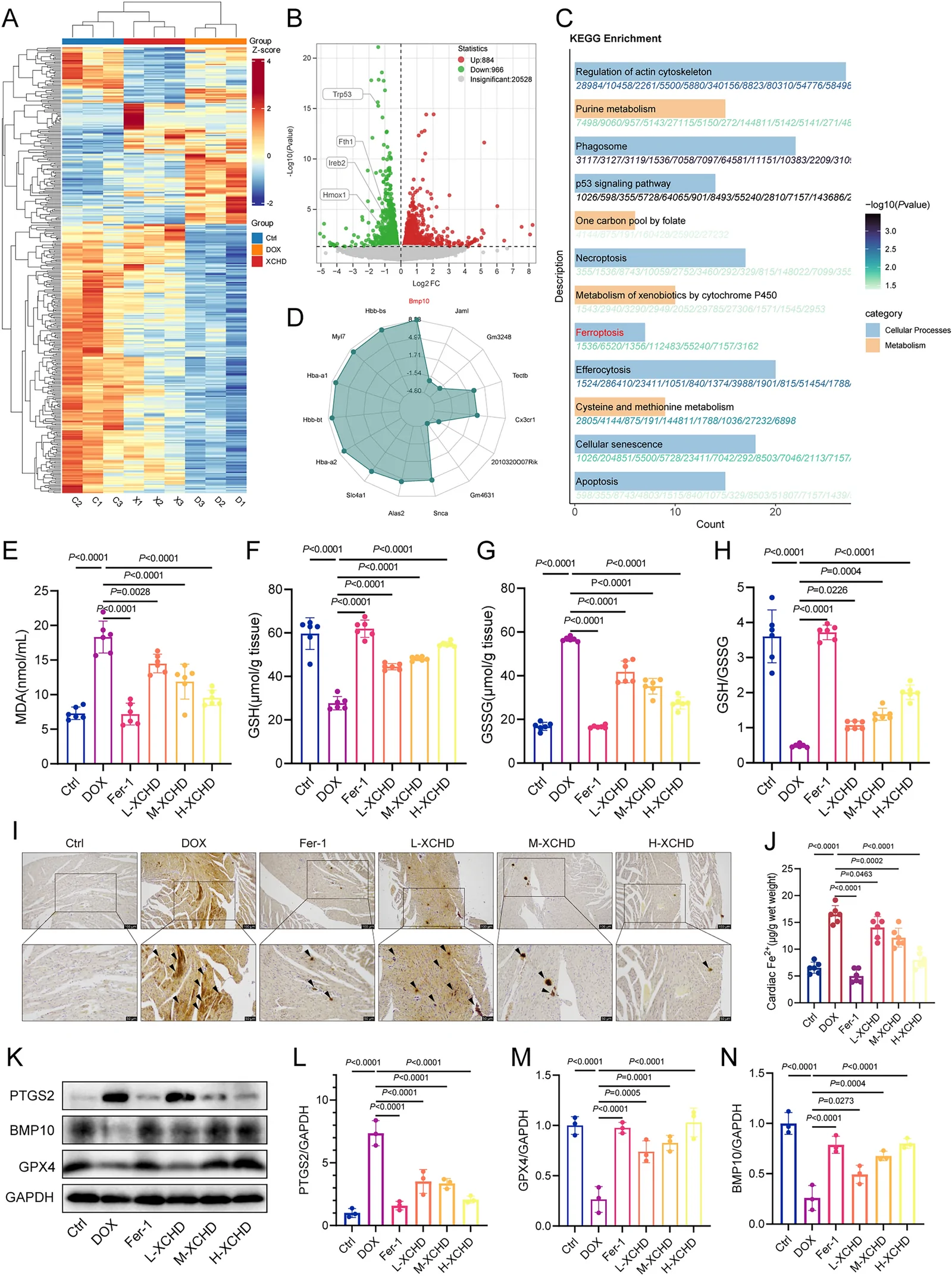
XCHD suppresses ferroptosis and restores BMP10 expression in cardiac tissue. **A** Heatmap of DEGs in Control, DOX, and XCHD groups (n = 3). **B** Volcano plot of DEGs between XCHD and DOX groups. Red: upregulated; green: downregulated. **C** KEGG enrichment highlighting the ferroptosis pathway. **D** Radar chart of top-ranked core genes, with Bmp10 identified as a prominent XCHD-upregulated target. **E** Myocardial MDA content (n = 6). **F**–**H** Myocardial GSH (**F**), GSSG (**G**), and GSH/GSSG ratio (**H**) (n = 6). **I** Representative Prussian blue staining of myocardial iron deposition (scale bar = 50 μm; n = 6). **J** Quantification of cardiac Fe^2^⁺ content (n = 6). **K** Representative Western blot of PTGS2, BMP10, GPX4, and GAPDH. **L**–**N** Densitometric quantification of PTGS2 (**L**), GPX4 (**M**), and BMP10 (**N**), normalized to GAPDH (n = 3). Data are expressed as mean ± SD. Statistical analysis was performed using one-way ANOVA followed by Dunnett's post hoc test. *P* values are indicated in the figures

To investigate the molecular basis of XCHD-mediated cardioprotection, transcriptomic profiling was performed using myocardial tissues. Hierarchical clustering analysis revealed distinct transcriptomic patterns among the groups, with the expression profile of the XCHD-treated group shifting toward that of the control group (Fig. 2A). Differential expression analysis identified 1,850 differentially expressed genes, including 884 upregulated and 966 downregulated genes (Fig. 2B).

To identify pathways potentially involved in DOX-induced cardiotoxicity and the effects of XCHD, KEGG enrichment analysis was performed. Ferroptosis was among the significantly enriched pathways (Fig. 2C), suggesting that ferroptosis-related processes may be involved in the cardioprotective effects of XCHD. Analysis of genes associated with this pathway identified BMP10 as a candidate gene that was suppressed by DOX and restored by XCHD (Fig. 2D). Although BMP10 is best known for its role in cardiac development and maintenance, recent studies have implicated it in cardiac iron homeostasis, with BMP10 deficiency promoting myocardial iron accumulation[18]. These findings provided a rationale for examining the potential association between BMP10 expression and ferroptosis-related alterations.

Consistent with the transcriptomic findings, DOX challenge induced ferroptosis-associated alterations in the heart, as indicated by increased MDA levels and a reduced GSH/GSSG ratio (Fig. 2E-H). Prussian blue staining showed increased iron deposition in the myocardium of DOX-treated mice (Fig. 2I), accompanied by elevated cardiac Fe^2^⁺ levels (Fig. 2J). XCHD treatment attenuated these abnormalities, including lipid peroxidation, redox imbalance, and iron accumulation. Western blot analysis further showed that DOX increased PTGS2 expression and decreased GPX4 expression, whereas XCHD reversed these changes (Fig. 2K-M). Consistent with the transcriptomic results, myocardial BMP10 protein expression was reduced following DOX treatment and restored by XCHD (Fig. 2N).

Taken together, these findings indicate that XCHD attenuates DOX-induced cardiac injury and ferroptosis-associated alterations in vivo, including lipid peroxidation, redox imbalance, iron accumulation, and changes in PTGS2 and GPX4 expression. Restoration of myocardial BMP10 expression accompanied these effects, supporting a potential association between BMP10 and ferroptosis-related processes during XCHD-mediated cardioprotection. This possibility was further examined in subsequent experiments.

### BMP10 knockdown weakens the anti-ferroptotic effects of XCHD-containing serum in H9c2 cells

**Fig. 3:**
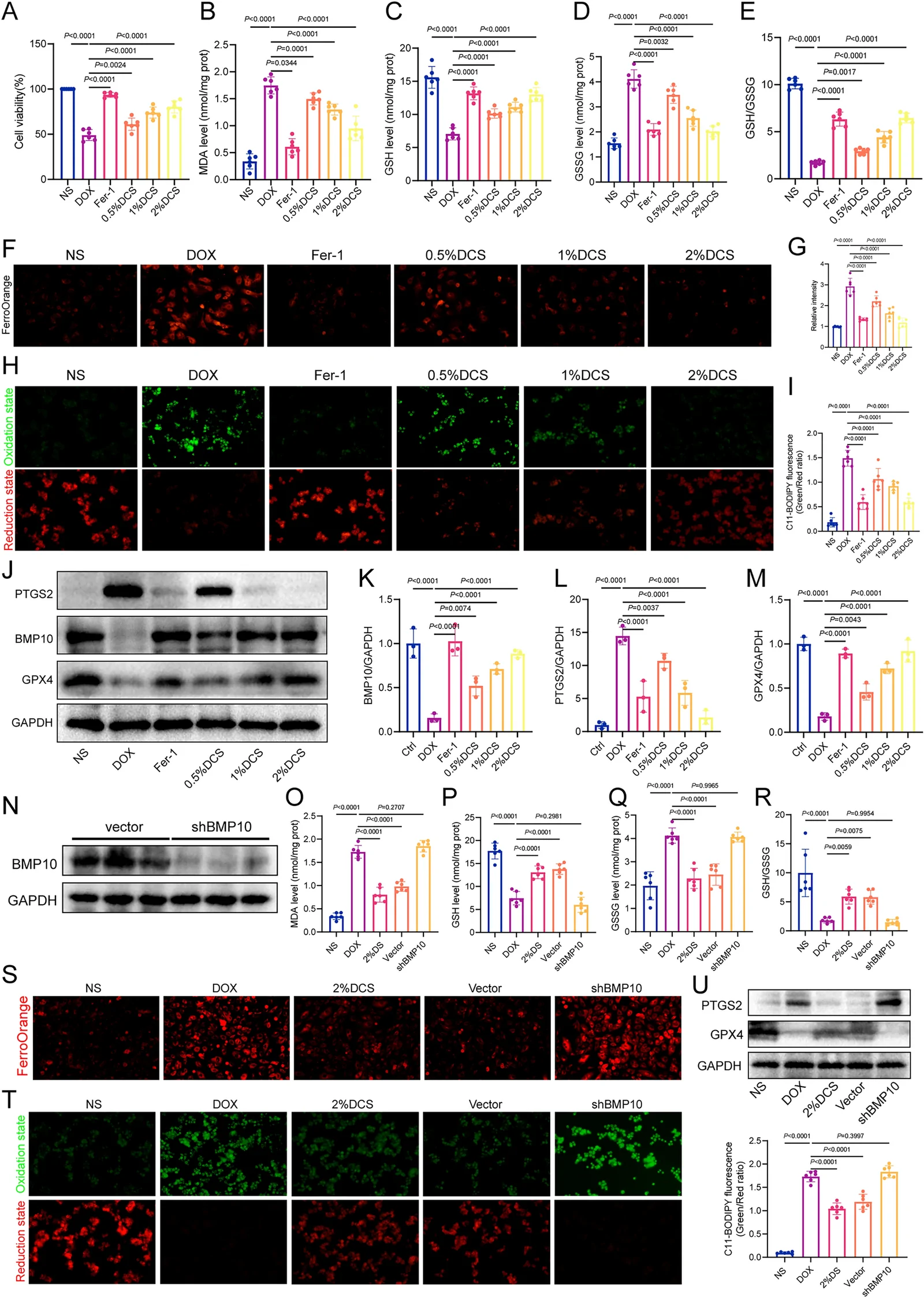
BMP10 knockdown weakens the anti-ferroptotic effects of XCHD-containing serum in H9c2 cells. **A** Cell viability following DOX ± DCS or Fer-1 treatment, assessed by CCK-8 (n = 6). **B**–**E** Intracellular redox parameters: MDA (**B**), GSH (**C**), GSSG (**D**), GSH/GSSG ratio (**E**) (n = 6). **F**–**G** Intracellular Fe^2^⁺ levels: representative FerroOrange images (**F**) and fluorescence quantification (**G**) (n = 6). **H**, **I** Oxidized/reduced C11-BODIPY fluorescence and ratio (n = 6). **J**–**M** Western blot of BMP10 (**K**), PTGS2 (**L**), and GPX4 (**M**) with GAPDH normalization (n = 3). **N** BMP10 knockdown validation by western blot (n = 3). **O**–**R** Effects of BMP10 silencing on MDA, GSH, GSSG, and GSH/GSSG ratio (n = 6). **S**, **T** Representative FerroOrange and C11-BODIPY images in BMP10-silenced cells. **U** PTGS2 and GPX4 expression in BMP10-silenced cells (n = 3). Data are expressed as mean ± SD. Statistical analysis was performed using one-way ANOVA followed by Dunnett's post hoc test. *P* values are indicated in the figures

Building on the in vivo transcriptomic findings, we next examined whether BMP10 contributes to the anti-ferroptotic effects of DCS in a cellular model of DOX-induced injury. H9c2 cells were exposed to DOX in the presence or absence of DCS. DOX treatment markedly reduced cell viability, whereas DCS improved cell viability in a concentration-dependent manner (Fig. 3A). DOX exposure also induced ferroptosis-associated alterations, including increased MDA levels, intracellular Fe^2^⁺ accumulation, lipid ROS generation, and disruption of glutathione redox homeostasis, as indicated by a reduced GSH/GSSG ratio. DCS attenuated these alterations, reduced iron-dependent lipid peroxidation, and restored cellular redox balance (Fig. 3B-I). Consistent with the in vivo observations, DCS restored BMP10 and GPX4 expression and reduced DOX-induced PTGS2 upregulation (Fig. 3J-M).

To determine whether BMP10 contributes to these effects, BMP10 expression was silenced using lentiviral shRNA (Fig. 3N). BMP10 knockdown substantially weakened the protective effects of DCS against DOX-induced injury. Specifically, the ability of DCS to reduce lipid peroxidation, intracellular iron accumulation, and ROS generation was diminished following BMP10 knockdown. BMP10 silencing also attenuated DCS-mediated restoration of GPX4 expression and suppression of PTGS2 upregulation (Fig. 3O-U). Together, these findings support a functional contribution of BMP10 to the anti-ferroptotic effects of DCS in H9c2 cells.

### XCHD preserves intestinal barrier integrity and remodels the gut microbiota with enrichment of *Akkermansia muciniphila*

**Fig. 4:**
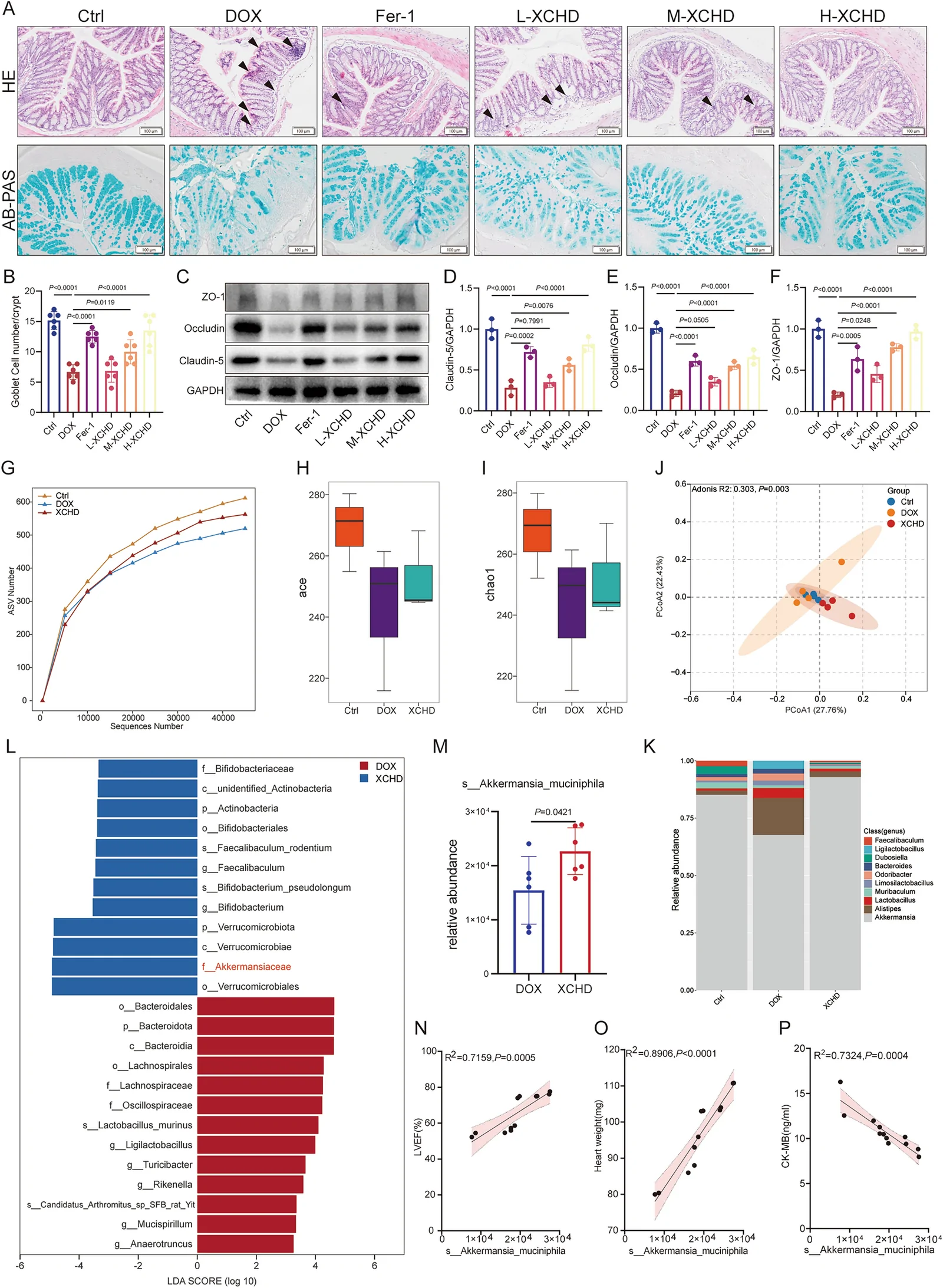
XCHD restores intestinal barrier integrity and selectively enriches *Akkermansia muciniphila* within the gut microbiota. **A** Representative H&E (top) and AB-PAS staining(bottom) of colon sections from the Ctrl, DOX, Fer-1, L-XCHD, M-XCHD, and H-XCHD groups (scale bars = 100 μm; n = 6). **B** Quantification of goblet cell number per crypt (n = 6). **C** Representative Western blot of colonic ZO-1, Occludin, Claudin-5, and GAPDH. **D**–**F** Densitometric quantification of Claudin-5 (**D**), Occludin (**E**), and ZO-1 (**F**), normalized to GAPDH (n = 3). **G** Rarefaction curves of observed OTUs in each group. **H**, **I** Alpha diversity indices: ACE (**H**) and Chao1 (**I**) (n = 4). **J** PCoA plot based on Bray–Curtis dissimilarity (Adonis R^2^ = 0.303, *P* = 0.003). **K** Genus-level relative abundance among Ctrl, DOX, and XCHD groups. **L** LEfSe analysis of differentially abundant taxa between DOX and XCHD groups (LDA > 2); the family Akkermansiaceae is highlighted in red. **M** Relative fecal abundance of AKK in DOX vs. XCHD groups (n = 6). **N**–**P** Pearson correlation analyses between fecal AKK abundance and LVEF (**N**), heart weight (**O**), and serum CK-MB (**P**). Data are presented as mean ± SD or as box plots. Statistical analysis was performed using one-way ANOVA followed by Dunnett's post hoc test for multi-group comparisons, unpaired Student's t-test for two-group comparisons, and Pearson's correlation analysis for correlations. *P* values are indicated in the figures

Given the involvement of the gut–heart axis in DOX-induced cardiotoxicity[28], we next investigated whether XCHD affects intestinal barrier integrity and gut microbiota composition. H&E and AB-PAS staining showed that DOX induced epithelial damage and goblet cell loss, both of which were progressively improved by XCHD, with the H-XCHD group showing effects comparable to those of Fer-1 (Fig. 4A, B). Consistently, DOX markedly reduced the expression of claudin-5, occludin, and ZO-1, whereas XCHD restored their expression in a dose-dependent manner (Fig. 4C-F). These findings prompted us to further examine the associated changes in the gut microbiota.

16S rRNA gene sequencing was subsequently performed using the H-XCHD group, which exhibited the most pronounced cardioprotective effects among the three XCHD doses. Rarefaction analysis confirmed sufficient sequencing depth (Fig. 4G). DOX reduced microbial richness, as reflected by decreased ACE and Chao1 indices, whereas XCHD partially restored microbial richness (Fig. 4H, I). PCoA showed a clear separation between the DOX and control groups, whereas the XCHD group clustered closer to the control group (Fig. 4J). At the phylum level, DOX reduced the relative abundance of Verrucomicrobiota and increased the relative abundances of Firmicutes and Bacteroidota, whereas XCHD largely reversed these changes and normalized the Firmicutes/Bacteroidota ratio (Supplementary Figure S2).

Genus-level analysis showed that DOX markedly depleted *Akkermansia* and increased the abundance of *Bacteroides*, *Ligilactobacillus*, *Dubosiella*, and *Odoribacter*, whereas XCHD shifted the microbial profile toward that of the control group (Fig. 4K). Linear discriminant analysis effect size (LEfSe) analysis comparing the DOX and XCHD groups identified distinct microbial signatures (Fig. 4L). XCHD preferentially enriched Verrucomicrobiota-related taxa, including AKK, as well as putatively beneficial genera such as *Bifidobacterium* and *Faecalibaculum.* Notably, AKK was significantly enriched in the XCHD group compared with the DOX group (Fig. 4M). Correlation analysis further showed that the abundance of AKK was positively associated with LVEF and heart weight and negatively associated with serum CK-MB levels (Fig. 4N-P).

Collectively, these findings indicate that XCHD alleviates DOX-induced gut microbial dysbiosis and preferentially enriches AKK, which may contribute to the modulation of the gut–heart axis.

### Gut microbiota depletion abolishes XCHD-mediated cardioprotection

**Fig. 5:**
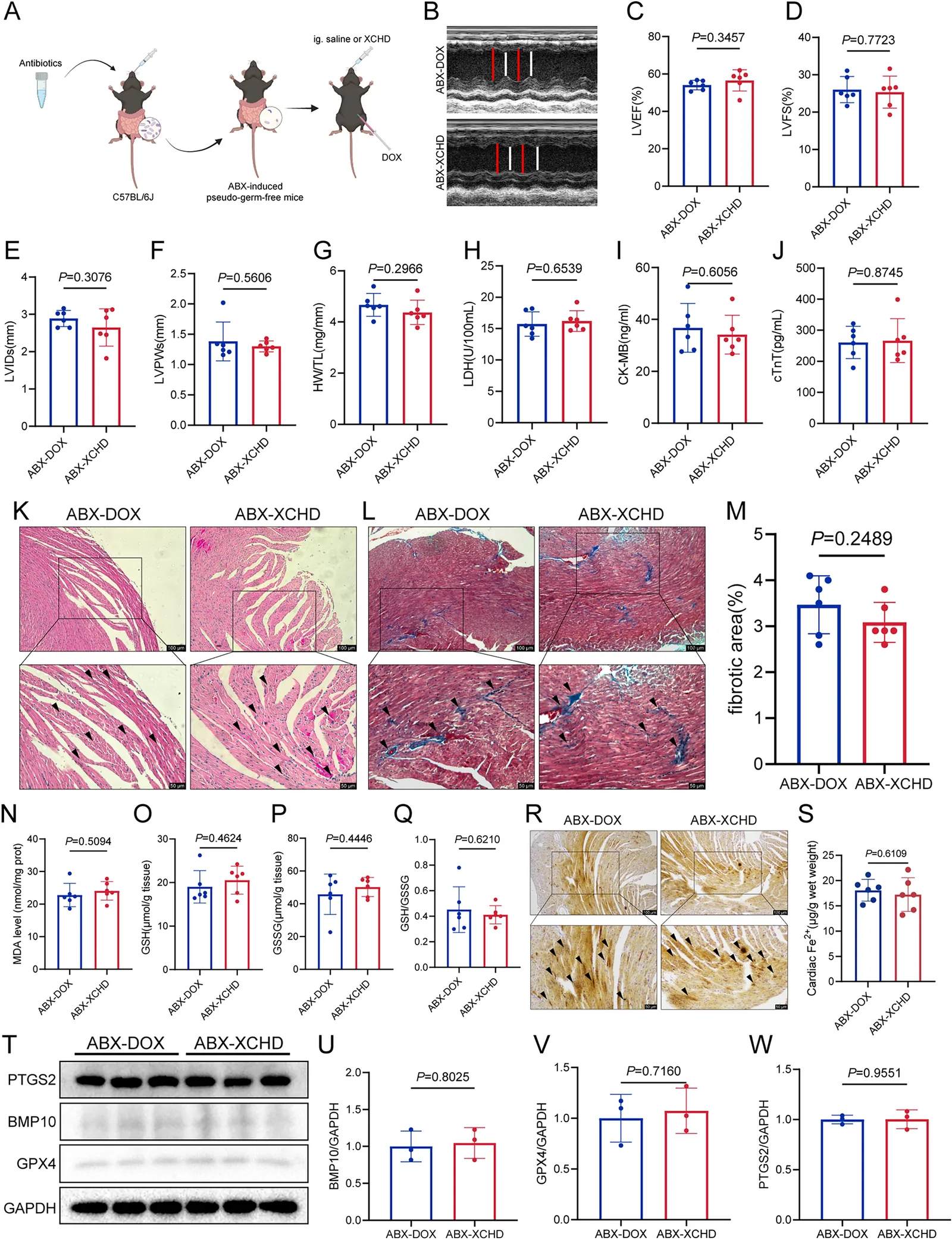
Gut microbiota depletion abolishes the cardioprotective effects of XCHD against DOX-induced cardiotoxicity. **A** Experimental schematic. Antibiotic-treated pseudo-germ-free mice received DOX with or without XCHD co-treatment. **B** Representative M-mode echocardiographic images. **C**–**F** LVEF (**C**), LVFS (**D**), LVIDs (**E**), and LVPWs (**F**) (n = 6). **G** HW/TL ratio (n = 6). **H**–**J** Serum LDH (**H**), CK-MB (**I**), and cTnT (**J**) (n = 6). **K**, **L** Representative H&E (**K**) and Masson's trichrome (**L**) staining of myocardial sections (scale bars = 50 μm; n = 6). **M** Quantification of fibrotic area (n = 6). **N**–**Q** Myocardial MDA (N), GSH (**O**), GSSG (**P**), and GSH/GSSG ratio (**Q**) (n = 6). **R** Representative Prussian blue staining of myocardial iron deposition (scale bar = 50 μm; n = 6). **S** Quantification of cardiac Fe^2^⁺ content (n = 6). **T** Representative Western blot of PTGS2, BMP10, GPX4, and GAPDH. **U**–**W** Densitometric quantification of BMP10 (**U**), GPX4 (**V**), and PTGS2 (**W**), normalized to GAPDH (n = 3). Data are presented as mean ± SD. Comparisons between two groups were performed using the unpaired Student's t-test. *P* values are indicated in the figures

To determine whether the gut microbiota contributes to XCHD-mediated cardioprotection, a PGF mouse model was established by broad-spectrum antibiotic treatment prior to DOX administration (Fig. 5A). Echocardiographic analysis showed that microbiota depletion abolished the beneficial effects of XCHD on cardiac function. In ABX-treated mice, XCHD failed to improve LVEF, LVFS, LVIDs, or LVPWs, and these parameters were comparable between the ABX-XCHD and ABX-DOX groups (Fig. 5B-F). Similarly, the reduced HW/TL ratio was not restored by XCHD treatment (Fig. 5G). Microbiota depletion also abolished the protective effects of XCHD against myocardial injury and remodeling. Serum levels of LDH, CK-MB, and cTnT remained elevated in ABX-XCHD mice and did not differ from those in ABX-DOX mice (Fig. 5H-J). Histological analyses likewise revealed comparable myocardial disorganization and interstitial fibrosis in the two groups (Fig. 5K-M).

The effects of XCHD on ferroptosis-associated alterations were also lost following microbiota depletion. XCHD no longer reduced myocardial MDA levels or restored the GSH/GSSG ratio in ABX-treated mice (Fig. 5N-Q). Prussian blue staining showed similar iron deposition in the ABX-DOX and ABX-XCHD groups (Fig. 5R), accompanied by persistently elevated cardiac Fe^2^⁺ levels (Fig. 5S). Western blot analysis further showed that microbiota depletion abolished the effects of XCHD on BMP10, GPX4, and PTGS2 expression (Fig. 5T-W). In addition, XCHD failed to improve colonic morphology, goblet cell abundance, or the expression of ZO-1, occludin, and claudin-5 in microbiota-depleted mice (Supplementary Figure S3), suggesting that the beneficial effects of XCHD on intestinal barrier integrity also depend on the presence of the gut microbiota.

Taken together, these findings indicate that depletion of the gut microbiota abolishes the cardioprotective effects of XCHD and its ability to attenuate ferroptosis-associated alterations, supporting the involvement of the gut–heart axis in the actions of XCHD.

### Fecal microbiota transplantation recapitulates the cardioprotective effects of Xiao Chai Hu Decoction

**Fig. 6:**
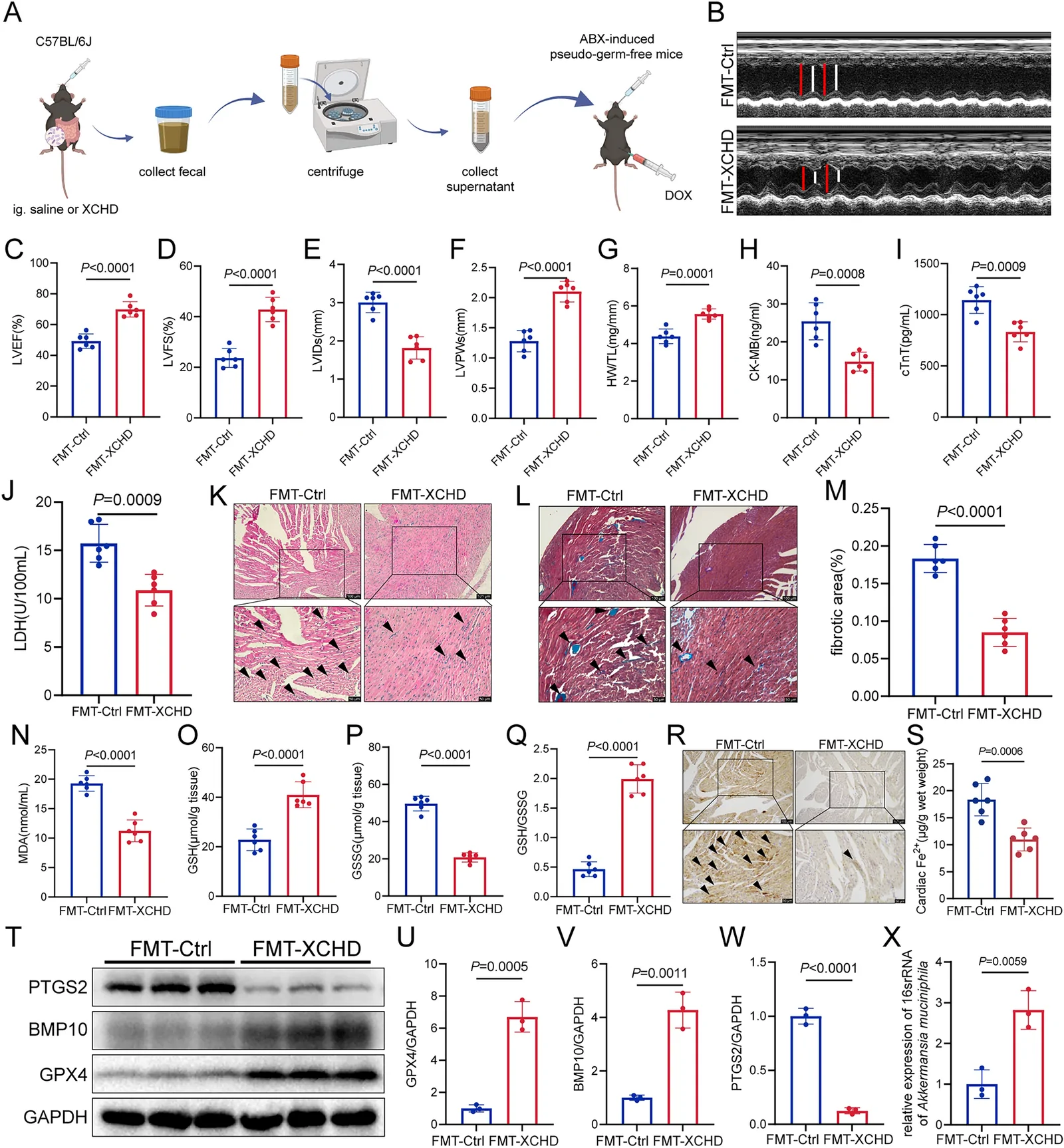
FMT from XCHD-treated mice confers cardioprotection against DOX-induced cardiotoxicity. **A** FMT experimental design. ABX-treated mice received fecal supernatants from Ctrl or XCHD donors, followed by DOX. **B** Representative M-mode images of the left ventricle. **C**–**F** LVEF (**C**), LVFS (**D**), LVIDs (**E**), and LVPWs (**F**) (n = 6). **G** HW/TL ratio (n = 6). **H**–**J** Serum CK-MB (**H**), cTnT (**I**), and LDH (**J**) (n = 6). **K**, **L** Representative H&E (**K**) and Masson's trichrome (**L**) staining of myocardial sections (scale bars = 50 μm; n = 6). **M** Quantification of fibrotic area (n = 6). **N**–**Q** Myocardial MDA (**N**), GSH (**O**), GSSG (**P**), and GSH/GSSG ratio (**Q**) (n = 6). **R** Representative Prussian blue staining of myocardial iron deposition (scale bar = 50 μm; n = 6). **S** Quantification of cardiac Fe^2^⁺ content (n = 6). **T** Representative Western blot of PTGS2, BMP10, GPX4, and GAPDH. **U**–**W** Densitometric quantification of GPX4 (**U**), BMP10 (**V**), and PTGS2 (**W**), normalized to GAPDH (n = 3). **X** Relative fecal abundance of AKK assessed by RT–qPCR (n = 3). Data are presented as mean ± SD. Comparisons between two groups were performed using the unpaired Student's t-test.* P* values are indicated in the figures

To investigate whether gut microbiota remodeling contributes to the cardioprotective effects of XCHD, FMT was performed in PGF recipient mice (Fig. 6A). Recipient mice received fecal microbiota from saline-treated (FMT-Ctrl) or XCHD-treated (FMT-XCHD) donors prior to DOX administration.

Echocardiographic analysis showed that FMT from XCHD-treated donors significantly improved cardiac function. Compared with FMT-Ctrl mice, FMT-XCHD recipients exhibited higher LVEF, LVFS, and LVPWs, together with lower LVIDs (Fig. 6B–F). HW/TL was also increased in FMT-XCHD mice (Fig. 6G). Consistently, serum levels of CK-MB, cTnT, and LDH were significantly reduced in FMT-XCHD recipients (Fig. 6H-J). Histological analyses further revealed improved myocardial architecture and reduced interstitial fibrosis, as shown by H&E and Masson’s trichrome staining (Fig. 6K-M).

FMT from XCHD-treated donors also attenuated ferroptosis-associated alterations. FMT-XCHD mice exhibited reduced MDA levels and an increased GSH/GSSG ratio compared with FMT-Ctrl mice (Fig. 6N-Q). Prussian blue staining showed decreased myocardial iron deposition in FMT-XCHD recipients, accompanied by lower cardiac Fe^2^⁺ levels (Fig. 6R-S). Western blot analysis showed increased BMP10 and GPX4 expression and decreased PTGS2 expression in FMT-XCHD mice relative to FMT-Ctrl mice (Fig. 6T-W). In addition, RT-qPCR analysis confirmed a higher fecal abundance of AKK in FMT-XCHD recipients (Fig. 6X), consistent with the transfer of XCHD-associated AKK enrichment. Consistent with the cardiac findings, FMT-XCHD mice exhibited improved colonic morphology, increased goblet cell abundance, and enhanced expression of ZO-1, occludin, and claudin-5 compared with FMT-Ctrl mice (Supplementary Figure S4), suggesting that gut microbiota remodeling contributes to the beneficial effects of XCHD on intestinal barrier integrity.

Collectively, these findings show that transplantation of microbiota from XCHD-treated donors recapitulates XCHD-mediated cardioprotection and attenuation of ferroptosis-associated alterations, supporting a role for gut microbiota remodeling in the effects of XCHD against DOX-induced cardiotoxicity.

### *Akkermansia muciniphila* attenuates doxorubicin-induced cardiotoxicity and ferroptosis-associated alterations

**Fig. 7:**
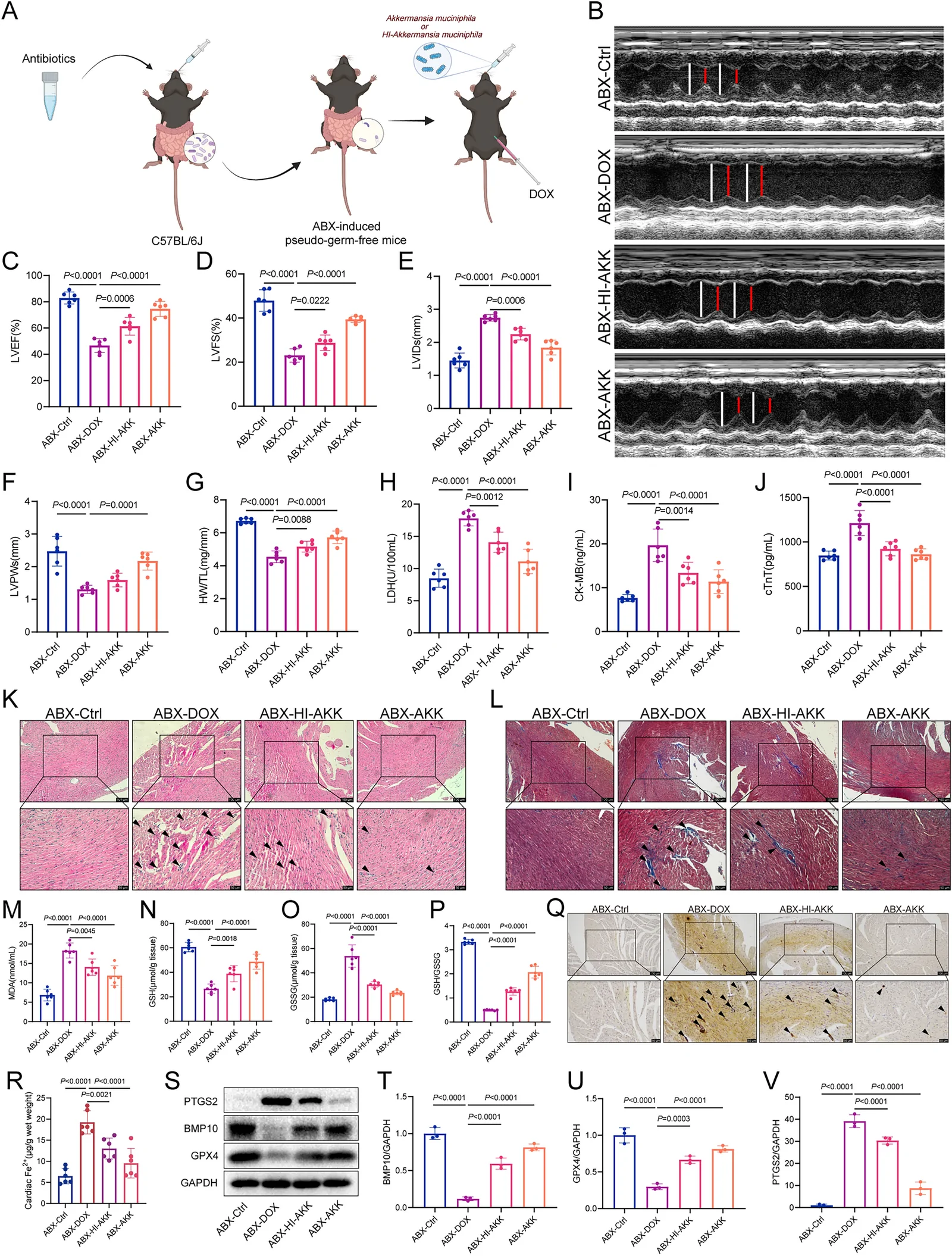
*Akkermansia muciniphila* attenuates DOX cardiotoxicity in a viability-dependent manner. **A** Experimental schematic. ABX-treated mice colonized with live or heat-inactivated AKK during DOX treatment. **B** Representative M-mode images of the left ventricle. **C**–**F** LVEF (**C**), LVFS (**D**), LVIDs (**E**), and LVPWs (**F**) (n = 6). **G** HW/TL ratio (n = 6). **H**–**J** Serum LDH (**H**), CK-MB (**I**), and cTnT (**J**) (n = 6). **K**, **L** Representative H&E (**K**) and Masson's trichrome (**L**) staining of myocardial sections (scale bars = 50 μm; n = 6). **M**–**P** Myocardial MDA (**M**), GSH (**N**), GSSG (**O**), and GSH/GSSG ratio (P) (n = 6). **Q** Representative Prussian blue staining of myocardial iron deposition (scale bar = 50 μm; n = 6). **R** Quantification of cardiac Fe^2^⁺ content (n = 6). **S** Representative Western blot of BMP10, GPX4, PTGS2, and GAPDH. **T**–**V** Densitometric quantification of BMP10 (**T**), GPX4 (**U**), and PTGS2 (**V**), normalized to GAPDH (n = 3). Data are expressed as mean ± SD. Statistical analysis was performed using one-way ANOVA followed by Dunnett's post hoc test. *P* values are indicated in the figures

To determine whether AKK contributes to cardioprotection and whether bacterial viability influences its effects, ABX-treated mice challenged with DOX received either live AKK (ABX-AKK) or HI-AKK (ABX-HI-AKK) (Fig. 7A).

Echocardiographic analysis revealed greater cardioprotective effects of live AKK than HI-AKK. Although both interventions partially improved DOX-induced cardiac dysfunction, live AKK produced greater improvements in LVEF, LVFS, LVIDs, and LVPWs than HI-AKK (Fig. 7B-F). Consistently, the HW/TL ratio was increased to a greater extent in the ABX-AKK group (Fig. 7G). Similar trends were observed for myocardial injury and histopathological changes. Serum levels of LDH, CK-MB, and cTnT were reduced in both groups, with greater reductions in mice receiving live AKK (Fig. 7H-J). Histological analyses further showed improved myocardial architecture and reduced interstitial fibrosis, with more pronounced effects in the ABX-AKK group (Fig. 7K, L).

Ferroptosis-associated alterations also differed according to bacterial viability. Compared with HI-AKK, live AKK more effectively reduced MDA levels and increased the GSH/GSSG ratio (Fig. 7M-P). Prussian blue staining and cardiac Fe^2^⁺ quantification further demonstrated lower myocardial iron accumulation in the ABX-AKK group (Fig. 7Q-R). Western blot analysis showed greater increases in BMP10 and GPX4 expression and a greater reduction in PTGS2 expression in mice receiving live AKK (Fig. 7S-V). Both live AKK and HI-AKK partially improved colonic morphology, goblet cell abundance, and the expression of ZO-1, occludin, and claudin-5, with more pronounced improvements observed in the ABX-AKK group (Supplementary Figure S5).

Taken together, these findings indicate that AKK attenuates DOX-induced cardiotoxicity and ferroptosis-associated alterations, with live AKK exerting greater protective effects than HI-AKK.

## Discussion

**Fig. 8:**
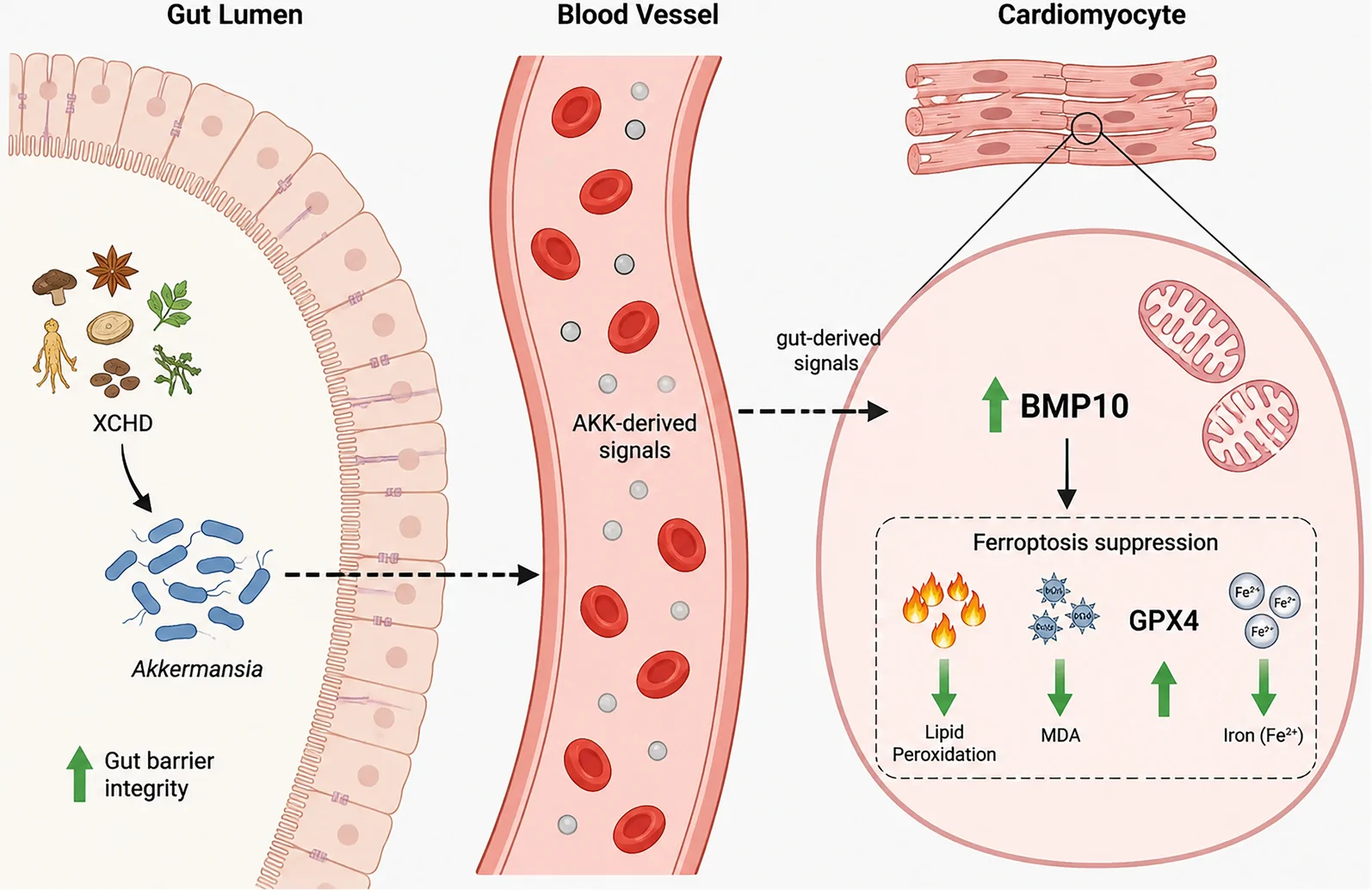
Proposed AKK-associated gut–heart mechanism underlying XCHD protection against DOX-induced cardiotoxicity. XCHD remodels the gut microbiota, enriches *Akkermansia muciniphila*, and preserves intestinal barrier integrity. These changes may influence the heart through currently unidentified circulating gut-derived signals. In the myocardium, XCHD-associated microbiota remodeling is accompanied by restoration of BMP10 expression, improved iron homeostasis, and attenuation of ferroptosis-associated alterations, including reduced lipid peroxidation, MDA production, and Fe^2^⁺ accumulation, together with increased GPX4 expression. These changes are associated with reduced DOX-induced myocardial injury. BMP10 knockdown weakened the anti-ferroptotic effects of XCHD-containing serum in H9c2 cells, supporting a downstream contribution of BMP10 in vitro. Solid arrows indicate experimentally supported relationships, whereas dashed arrows indicate proposed but unresolved links

DOX remains a cornerstone chemotherapeutic agent; however, its clinical utility is severely limited by cumulative and often irreversible cardiotoxicity. Current cardioprotective strategies largely focus on directly alleviating myocardial mitochondrial dysfunction but generally provide incomplete protection. Attention has therefore shifted toward upstream regulatory systems, among which the gut-heart axis has emerged as an important determinant of cardiovascular homeostasis [29, 30]. In this context, the present study supports a microbiota-dependent gut-heart mechanism underlying XCHD-mediated protection against DOX-induced cardiotoxicity, in which AKK enrichment is linked to myocardial BMP10 restoration and attenuation of ferroptosis-related injury (Fig. 8).

Compared with previous studies, the present work extends TCM-mediated cardioprotection from cardiomyocyte-intrinsic pathways to an upstream microbiota-dependent mechanism. Earlier studies have mainly focused on the direct regulation of oxidative stress, inflammation, apoptosis, or ferroptosis, whereas microbiota-related investigations have often remained descriptive or correlational. By combining ABX-mediated microbiota depletion, FMT, and AKK supplementation, we examined microbiota dependence, transferability of the protective phenotype, and the functional contribution of AKK, thereby supporting a “TCM formula-gut microbiota-host response-regulated cell death” framework for XCHD-mediated cardioprotection.

Our findings may also provide a biological perspective for interpreting the TCM concept that the “spleen and stomach” influence cardiac function within the modern framework of the gut-heart axis. Consistent with previous reports that DOX disrupts intestinal barrier integrity and promotes systemic inflammation [31], the ABX, FMT, and AKK intervention experiments collectively support a microbiota-dependent contribution to the intestinal and cardiac effects of XCHD. AKK was prioritized because XCHD increased its abundance, its abundance correlated with cardiac function, and supplementation reproduced key protective effects. Given its established role in maintaining mucosal homeostasis [32, 33], AKK may act as an upstream microbial regulator of pathological gut-heart signaling during DOX-induced cardiotoxicity.

Intestinal bacteria are unlikely to regulate myocardial BMP10 expression directly, suggesting that the observed association between AKK and BMP10 may depend on circulating intermediate signals. Two non-mutually exclusive mechanisms may be involved. First, metabolically active AKK may modify the availability of microbial metabolites, including short-chain fatty acids, bile acids, and tryptophan-derived metabolites, or release bioactive components such as Amuc_1100 and outer membrane vesicles [34]. These signals could influence myocardial BMP10 expression and susceptibility to ferroptosis through inflammatory, redox, and iron-homeostatic pathways [31, 35, 36]. Second, reinforcement of the mucus layer and epithelial tight junctions may limit microbial product translocation, thereby reducing systemic inflammation and oxidative stress and indirectly favoring myocardial BMP10 recovery. The stronger effects of live AKK than HI-AKK suggest a contribution from metabolically active processes, whereas the partial protection afforded by HI-AKK indicates that thermostable bacterial components may also participate. Because these intermediate pathways were not directly examined, these possibilities remain hypothetical rather than established causal mechanisms.

At the molecular level, BMP10 may represent a cardiac downstream node of gut-derived signaling rather than a direct target of intestinal bacteria. Although BMP10 has traditionally been recognized as a developmental ligand [37], emerging evidence suggests that it also contributes to adult cardiac homeostasis. Across XCHD treatment, FMT, AKK supplementation, and microbiota depletion, changes in myocardial BMP10 expression consistently paralleled ferroptosis-related markers and cardiac phenotypes. Together with the H9c2 knockdown results, these findings support BMP10 as a downstream contributor to the anti-ferroptotic effects of XCHD-containing serum in vitro. However, they do not establish that BMP10 is required for the cardioprotective effects of FMT or AKK in vivo. Moreover, the circulating signal responsible for BMP10 regulation and the precise molecular mechanism controlling its expression remain to be determined.

Several limitations of the present study should be acknowledged. First, the relative contribution of ferroptosis compared with other forms of regulated cell death remains unclear, and cardiac-specific BMP10 loss-of-function and rescue studies are required to establish its in vivo necessity. H9c2 cells also do not fully recapitulate mature cardiomyocytes, although the in vitro observations were supported by corresponding findings in adult mouse hearts. Second, the circulating mediators linking AKK to myocardial BMP10 were not measured, precluding discrimination among directly absorbed XCHD constituents, microbiota-derived metabolites, and microbial biotransformation products. Integrated metabolomic, intestinal permeability, endotoxin, and inflammatory profiling, followed by supplementation, depletion, or receptor-blockade experiments, will be needed to identify the responsible mediators. The contributions of other XCHD-responsive microbial taxa also remain unresolved. Finally, the present study was conducted in non-tumor-bearing mice. Further studies using tumor-bearing models are required to determine whether XCHD provides cardioprotection without interfering with the antitumor efficacy of DOX.

## Conclusion

In conclusion, XCHD alleviates DOX-induced cardiotoxicity through an AKK-associated, microbiota-dependent gut–heart mechanism involving intestinal barrier preservation and attenuation of ferroptosis-associated injury, accompanied by restoration of myocardial BMP10 expression. ABX depletion, FMT, and AKK supplementation support the microbiota-dependent component, whereas BMP10 knockdown supports a downstream role for BMP10 in vitro. These findings support a “TCM formula–gut microbiota–host response–regulated cell death” framework and provide a conceptual basis for microbiota-oriented strategies to mitigate DIC.

## Supplementary Information

Additional file 1.

## Data Availability

The datasets used and/or analysed during the current study are available from the corresponding author on reasonable request.

## Abbreviations

AKK: *Akkermansia muciniphila*
BMP10: Bone morphogenetic protein 10
CK-MB: Creatine Kinase-MB isoenzyme
cTnT: Cardiac Troponin T
DCS: XCHD-containing serum
DOX: Doxorubicin
FMT: Fecal microbiota transplantation
GPX4: Glutathione peroxidase 4
GSH/GSSG: Reduced glutathione/oxidized glutathione
HI-AKK: Heat-inactivated *Akkermansia muciniphila*
LDH: Lactate dehydrogenase
LVEF: Left ventricular ejection fraction
LVFS: Left ventricular fractional shortening
LVIDs: Left ventricular internal diameter at end-systole
LVPWs: Left ventricular posterior wall thickness at end-systole
MDA: Malondialdehyde
NS: Normal serum
PGF: Pseudo-germ-free
PTGS2: Prostaglandin-endoperoxide synthase 2
TCM: Traditional Chinese medicine
XCHD: Xiao Chai Hu Decoction
