# Supplementary material for: Xiao Chai Hu Decoction ameliorates doxorubicin-induced cardiotoxicity through an Akkermansia muciniphila–associated gut–heart mechanism
Source: Chin Med. 2026 Nov 14;21:240. doi: 10.1186/s13020-026-01531-9 (PMC13769180; doi:10.1186/s13020-026-01531-9)
Supplement: Supplementary file 1 [file 13020_2026_1531_MOESM1_ESM.docx]

**Supplementary Table S1. Bioactive compounds consistently identified in both the Xiao Chai Hu Decoction (XCHD) decoction and the XCHD-containing serum by UPLC-MS/MS.**

| **No.** | **RT (min)** | **Metabolite** | **Mode** | **Molecular Formula** | ***m/z*** | **Chemical Class** |
| --- | --- | --- | --- | --- | --- | --- |
| 1 | 8.74 | Formononetin | neg | C₁₆H₁₂O₄ | 267.0665 | Isoflavonoid |
| 2 | 6.73 | Liquiritigenin | pos | C₁₅H₁₂O₄ | 257.0800 | Flavanone |
| 3 | 4.13 | Asperulosidic acid | neg | C₁₈H₂₄O₁₂ | 467.0974 | Iridoid glycoside |
| 4 | 10.49 | Platycodigenin | pos | C₃₀H₄₈O₇ | 485.3249 | Triterpenoid saponin aglycone |
| 5 | 5.82 | Vitexin | pos | C₂₁H₂₀O₁₀ | 433.1117 | Flavone C-glycoside |
| 6 | 6.30 | Scopoletin | neg | C₁₀H₈O₄ | 191.0356 | Coumarin |
| 7 | 11.34 | Polygalacic acid | pos | C₃₀H₄₈O₆ | 487.3406 | Triterpenoid saponin aglycone |
| 8 | 5.77 | Naringenin chalcone | pos | C₁₅H₁₂O₅ | 273.0748 | Chalcone |
| 9 | 6.83 | Emodin-8-glucoside | neg | C₂₁H₂₀O₁₀ | 431.0996 | Anthraquinone glycoside |
| 10 | 12.52 | 2,3,23-Trihydroxy-12-oleanen-28-oic acid | neg | C₃₀H₄₈O₅ | 469.3333 | Oleanane triterpenoid |
| 11 | 11.76 | Ilexgenin A | neg | C₃₀H₄₆O₆ | 483.3122 | Triterpenoid |
| 12 | 12.49 | 16α-Hydroxydehydrotrametenolic acid | pos | C₃₀H₄₆O₄ | 471.3457 | Lanostane triterpenoid |
| 13 | 1.58 | Inosine | neg | C₁₀H₁₂N₄O₅ | 267.0736 | Purine nucleoside |
| 14 | 6.30 | Quercitrin | neg | C₂₁H₂₀O₁₁ | 447.0946 | Flavonol glycoside |
| 15 | 6.76 | Aloenin | pos | C₁₉H₂₂O₁₀ | 433.1117 | Chromone glycoside |
| 16 | 10.95 | Gardenolic acid B | neg | C₃₀H₄₆O₅ | 485.3280 | Triterpenoid |
| 17 | 13.23 | Echinocystic acid | neg | C₃₀H₄₈O₄ | 471.3490 | Oleanane triterpenoid |
| 18 | 7.05 | 3-Epimeliasenin B | pos | C₃₀H₄₄O₄ | 469.3298 | Limonoid |
| 19 | 10.71 | 6α,16,18-Trihydroxycleroda-3,13-dien-15,16-olide | neg | C₂₀H₃₀O₅ | 331.1922 | Clerodane diterpenoid |
| 20 | 0.95 | Citrusin D | pos | C₁₆H₂₂O₈ | 360.1639 | Phenylpropanoid glycoside |
| 21 | 10.50 | 3α-Akebonoic acid | neg | C₂₉H₄₄O₃ | 485.3279 | Triterpenoid |
| 22 | 11.17 | Oleacein | pos | C₁₇H₂₀O₆ | 303.1217 | Secoiridoid |
| 23 | 5.83 | 4″-Methyloxy-genistin | neg | C₂₂H₂₂O₁₀ | 481.0913 | Isoflavone glycoside |
| 24 | 12.45 | Rubiarbonone E | neg | C₃₀H₄₆O₄ | 505.3092 | Triterpenoid |
| 25 | 10.44 | Kuguacin J | pos | C₃₀H₄₆O₃ | 419.3293 | Cucurbitane triterpenoid |
| 26 | 7.12 | Baicalin methyl ester | neg | C₂₂H₂₀O₁₁ | 459.0940 | Flavone glycoside |
| 27 | 6.84 | Pendulone | pos | C₁₇H₁₆O₆ | 299.0904 | Isoflavanquinone |
| 28 | 6.86 | Gossypetin 3-methyl ether | pos | C₁₆H₁₂O₈ | 315.0488 | Flavonol |
| 29 | 10.26 | Seconeokadsuranic acid A | pos | C₃₀H₄₄O₄ | 469.3296 | Triterpenoid |
| 30 | 5.31 | Isoengeletin | neg | C₂₁H₂₂O₁₀ | 433.1149 | Flavanonol glycoside |
| 31 | 8.87 | Cucurbitacin IIb | pos | C₃₀H₄₈O₇ | 485.3248 | Cucurbitane triterpenoid |

Compounds were identified by UPLC-MS/MS as previously described (Wang et al., J Ethnopharmacol 2024, DOI: 10.1016/j.jep.2024.118740). RT, retention time; pos, positive ion mode; neg, negative ion mode.

**Supplementary Figure S1. Determination of the appropriate serum concentration of normal serum (NS) and XCHD-containing serum (DCS) in H9C2 cells.**

(A) H9C2 cell viability following treatment with increasing concentrations (0.25–8%, v/v) of NS. (B) H9C2 cell viability following treatment with increasing concentrations (0.25–8%, v/v) of DCS. Data are presented as mean ± SD (n = 6). ****P < 0.0001.

**Supplementary Figure S2. XCHD partially reversed DOX-induced alterations in gut microbiota at the phylum level.**

(A) Relative abundance of the major bacterial phyla in the Ctrl, DOX, and XCHD groups. (B) Quantitative analysis of the Firmicutes/Bacteroidota (F/B) ratio. Data are presented as mean ± SD.

**Supplementary Figure S3. Gut microbiota depletion abolishes the protective effect of XCHD on intestinal barrier injury.**

(A) Representative H&E (top) and AB-PAS (bottom) staining. Arrowheads indicate epithelial damage. Scale bar = 100 μm. (B) Goblet cell number per crypt (n = 6 per group). (C) Representative immunoblots of ZO-1, Occludin, and Claudin-5. GAPDH served as the loading control. (D–F) Densitometric analysis of Claudin-5 (D), Occludin (E) and ZO-1 (F) normalized to GAPDH (n = 3 per group). Data are presented as mean ± SD. Unpaired two-tailed Student's t-test.

**Supplementary Figure S4. Microbiota transfer from XCHD-treated donors preserves intestinal barrier integrity in recipient mice.**

(A) Representative H&E (top) and AB-PAS (bottom) staining. Arrowheads indicate epithelial damage. Scale bar = 100 μm. (B) Goblet cell number per crypt (n = 6 per group). (C) Representative immunoblots of ZO-1, Occludin, and Claudin-5. GAPDH served as the loading control. (D–F) Densitometric analysis of Claudin-5 (D), Occludin (E), and ZO-1 (F) normalized to GAPDH (n = 3 per group). Data are presented as mean ± SD. Unpaired two-tailed Student's t-test.

**Supplementary Figure S5. Live AKK preserves intestinal barrier integrity in ABX-DOX mice.**

(A) Representative H&E (top) and AB-PAS (bottom) staining. Arrowheads indicate epithelial damage. Scale bar = 100 μm. (B) Goblet cell number per crypt (n = 6 per group). (C) Representative immunoblots of ZO-1, Occludin, and Claudin-5. GAPDH served as the loading control. (D–F) Densitometric analysis of Claudin-5 (D), Occludin (E), and ZO-1 (F) normalized to GAPDH (n = 3 per group). Data are presented as mean ± SD. One-way ANOVA followed by Dunnett's post hoc test.

Supplementary Figure S1

Supplementary Figure S2

Supplementary Figure S3

Supplementary Figure S4

Supplementary Figure S5
